# Trypanosomes Modify the Behavior of Their Insect Hosts: Effects on Locomotion and on the Expression of a Related Gene

**DOI:** 10.1371/journal.pntd.0003973

**Published:** 2015-08-20

**Authors:** Newmar Pinto Marliére, José Manuel Latorre-Estivalis, Marcelo Gustavo Lorenzo, David Carrasco, Juliana Alves-Silva, Juliana de Oliveira Rodrigues, Luciana de Lima Ferreira, Luisa de Melo Lara, Carl Lowenberger, Alessandra Aparecida Guarneri

**Affiliations:** 1 Centro de Pesquisas René Rachou, Avenida Augusto de Lima, Belo Horizonte, Minas Gerais, Brazil; 2 Chemical Ecology Group, Department of Plant Protection Biology, Swedish University of Agricultural Sciences, Alnarp, Sweden; 3 Departamento de Bioquímica e Imunologia, Instituto de Ciências Biológicas, Universidade Federal de Minas Gerais, Belo Horizonte, Minas Gerais, Brazil; 4 Department of Biological Sciences, Simon Fraser University, Burnaby, British Columbia, Canada; Universidad Autónoma de Yucatán, MEXICO

## Abstract

**Background:**

As a result of evolution, the biology of triatomines must have been significantly adapted to accommodate trypanosome infection in a complex network of vector-vertebrate-parasite interactions. Arthropod-borne parasites have probably developed mechanisms, largely still unknown, to exploit the vector-vertebrate host interactions to ensure their transmission to suitable hosts. Triatomines exhibit a strong negative phototaxis and nocturnal activity, believed to be important for insect survival against its predators.

**Methodology/Principal Findings:**

In this study we quantified phototaxis and locomotion in starved fifth instar nymphs of *Rhodnius prolixus* infected with *Trypanosoma cruzi* or *Trypanosoma rangeli*. *T*. *cruzi* infection did not alter insect phototaxis, but induced an overall 20% decrease in the number of bug locomotory events. Furthermore, the significant differences induced by this parasite were concentrated at the beginning of the scotophase. Conversely, *T*. *rangeli* modified both behaviors, as it significantly decreased bug negative phototaxis, while it induced a 23% increase in the number of locomotory events in infected bugs. In this case, the significant effects were observed during the photophase. We also investigated the expression of *Rpfor*, the triatomine ortholog of the *foraging* gene known to modulate locomotion in other insects, and found a 4.8 fold increase for *T*. *rangeli* infected insects.

**Conclusions/Significance:**

We demonstrated for the first time that trypanosome infection modulates the locomotory activity of the invertebrate host. *T*. *rangeli* infection seems to be more broadly effective, as besides affecting the intensity of locomotion this parasite also diminished negative phototaxis and the expression of a behavior-associated gene in the triatomine vector.

## Author Summary

The control of Chagas disease, an infection that affects ca. 8 million people in Latin America, is mostly based on vector control activities. Understanding vector biology and how these insects interact with their environment, hosts and pathogens is crucial to improve vector control strategies. The behavior of triatomines has been largely studied, yet few reports have focused on the behavioral effects of the interaction that these insects endure with their natural parasites. *Trypanosoma cruzi* and *Trypanosoma rangeli* are two protozoan parasites found naturally infecting *Rhodnius* species. In this study, we showed for the first time that the locomotory activity of *Rhodnius prolixus*, a relevant vector of Chagas disease, is affected by trypanosome infection. *T*. *cruzi* was found to decrease bug locomotory activity during night hours, while *T*. *rangeli* promoted a generally increased insect locomotion. In addition, we searched for the *R*. *prolixus* orthologue (*Rpfor*) of a gene associated with the modulation of insect activity (*foraging* gene) and found that *Rpfor* expression was also affected by trypanosome infection.

## Introduction

The triatomine bug *Rhodnius prolixus* (Hemiptera: Reduviidae) is an important vector of Chagas disease in Northern South America.

Parasites transmitted by blood-sucking insects are responsible for nearly 16% of the global burden of transmissible diseases [1] and their dispersal is highly dependent on the behavior of their arthropod vectors. Some vector-borne parasites have been shown to modify physiological and behavioral traits of both vectors and vertebrate hosts, in a way that increases the probability of transmission [2]. Identifying the underlying bases of these parasite-induced alterations is of great importance to understand disease biology which may lead to the design of new control measures [3].

*T*. *cruzi*, the causative agent of Chagas disease, is generally considered as non-pathogenic to triatomines because several previous works showed no pathological effects of this parasite to triatomines [4–6]. Nevertheless some other reports have shown costs induced by *T*. *cruzi* infections on its insect vectors [7–10]. A recent study published in our laboratory has clearly shown that *T*. *cruzi* infection decreases the fecundity and fertility of *R*. *prolixus* adults [10]. *Trypanosoma rangeli* is also transmitted by *Rhodnius* species and does not cause disease to humans, but its pathogenicity for triatomines has been confirmed by many authors [11–13]. Studies recently published by our laboratory have shown that infection with *T*. *rangeli* extends intermolt periods [14] and affects reproductive parameters in *R*. *prolixus* [14].

Triatomines present a bimodal pattern of daily activity, leaving their refuges when light intensity declines and displaying most of their activity during the first hours of the scotophase [15–17]. Before sunrise, they return to their refuges mainly guided by chemical signals [18]. During daylight hours, bugs remain aggregated inside these shelters and present low locomotory activity [17]. Different factors are considered to modulate the locomotion of insect vectors, e.g. *Culex annulirostris* varies its activity pattern as an adaptation to local climate [19]. The locomotory activity of *Cimex lectularius* is known to be controlled by nutritional status, as starved insects are less active than recently fed ones [20].

Alterations in locomotory activity induced by physiological changes may be mediated by modulation of gene expression and/or posttranscriptional mechanisms [21]. Interestingly, the sand fly *Lutzomyia longipalpis* shows a reduction in locomotory activity correlated with a downregulation in the expression of *period* and *timeless* genes after blood meals [22]. cGMP-dependent protein kinases (PKGs) are serine/threonine kinases [23] found in diverse organisms from paramecia to humans [24]. A PKG encoded by the gene named *foraging* was first reported to control the locomotory activity of *Drosophila melanogaster* [25]. The role of these proteins in modulating foraging behavior is highly conserved across species [26].

Recently our laboratory group has been investigating the effects of trypanosomes on their invertebrate hosts [10, 14, 27]. Understanding how pathogens modify activities such as locomotion will contribute to our understanding of pathogen transmission dynamics. In the present study two behavioral parameters were evaluated in *R*. *prolixus* nymphs infected with either *T*. *cruzi* or *T*. *rangeli*. We first evaluated the potential effects of parasites on the negative phototaxis of these insects, i.e., tested whether they have a weaker avoidance for illuminated places. Secondly, we developed an actometer to evaluate patterns of locomotory activity and characterized alterations induced by trypanosome infection. Finally, we characterized the expression levels of the *R*. *prolixus* PKG orthologue (*Rpfor*) in the brain of healthy and infected insects.

## Materials and Methods

### Ethics statement

All experiments using live animals were performed in accordance with FIOCRUZ guidelines on animal experimentation and were approved by the Ethics Committee in Animal Experimentation (CEUA/FIOCRUZ) under the approved protocol number L-058/08. This protocol adheres to the guidelines of CONCEA/MCT (http://www.cobea.org.br/), which is the maximum ethics committee of the Brazilian government.

### Experimental animals

*Rhodnius prolixus*: insects were obtained from a laboratory colony derived from insects collected in Honduras around 1990. The colony was maintained at 26±1°C, 50±5% RH and exposed to a natural illumination cycle. Insects were consistently fed on diverse sources of blood that included mice, chicken and a membrane feeder offering citrated rabbit blood at 37°C. Living hosts were anesthetized with an intraperitoneal injection of a ketamine (150 mg/kg; Cristália, Brazil)/xylazine (10 mg/kg; Bayer, Brazil) mixture. Fifth instar nymphs starved for 30 days after ecdysis were used in all the experiments.

### Parasites

*Trypanosoma cruzi* CL and *Trypanosoma rangeli* CHOACHI strains originally isolated from naturally infected *T*. *infestans* [28] and *R*. *prolixus* [29], respectively, were used in this study. The epimastigote forms used to infect insects were cultured by twice weekly passages at 27°C in liver-infusion tryptose (LIT) medium supplemented with 15% fetal bovine serum, 100 mg/ml of streptomycin and 100 units/ml of penicillin.

### Trypanosome infection

#### T. cruzi

*T*. *cruzi* infection was performed according to the methodology described before [30]. Second instar nymphs were allowed to feed on heat-inactivated rabbit blood containing culture epimastigotes (1×10^7^ parasites/ml) through a latex-membrane feeder at 37°C. Control insects were fed on blood to which a proportional dose of the sterile culture medium was added. Ten days after molting to the 3^rd^ instar, insects were fed on anaesthetized mice and immediately transferred to 1.5ml plastic tubes. To select insects for experiments with infected nymphs, the urine and feces released during their diuresis were examined for trypomastigotes and only infected insects were used in subsequent assays.

#### T. rangeli

The intracoelomatic infection of insects with *T*. *rangeli* was performed as described elsewhere [14]. Briefly, epimastigotes were obtained from 10 day old cultures, washed and resuspended in sterile PBS (0.15 M NaCl in 0.01 M sodium phosphate, pH 7.4, 2,000 rpm x 10min) in a concentration of 1x10^5^ par/ml. Seven day old 4^th^ instar nymphs were inoculated through the thoracic pleura with 1μl of the parasite suspension, using a 50μl syringe (Hamilton Company, USA, needle 13x3.3; ½'') connected to a dispenser (model 705, Hamilton Company, USA). Control insects were inoculated with the same volume of PBS. One day after infection, insects were fed on anaesthetized mice. To confirm trypanosome infection on insects, a sample of hemolymph was extracted by cutting the tarsus from one of the middle legs and examined under the microscope. The same procedure was carried out on control individuals to ensure a homogeneous manipulation of all experimental insects.

### Negative phototaxis

Negative phototaxis, i.e. the guidance reaction in which animals steer their way against light, allows the location of shelters and also helps the insect to avoid exposure to predators [31]. To study the phototactic responses of these *R*. *prolixus*, experiments were performed as described before [32]. A rectangular glass box (14×5×5cm) was divided lengthwise into two experimental arenas (2.5cm width each) and covered with a rectangular acrylic lid. The parallel design allowed the simultaneous evaluation of individual uninfected and infected insects. Half of each arena was maintained in the dark by a black piece of cardboard fixed on the cover of the box and the other half remained illuminated (190 lux). An initial batch of 70 nymphs (control = 35; infected = 35) was used in the assays that evaluated *T*. *cruzi* infection. For *T*. *rangeli* infections, 84 nymphs were used (control = 42; infected = 42). Insects that did not move during the trials were excluded from the analysis. Therefore, the analyses were performed comparing the responses of 34 control *vs* 28 *T*. *cruzi* infected bugs, and 42 control *vs* 39 *T*. *rangeli* infected bugs. In each trial, both nymphs were individually placed at one end of each arena inside small dark bowls (light and dark sides were alternated in subsequent assays). After 40s the bowls were removed and the trial started. Each trial lasted for 10min during which two behavioral parameters were measured, negative phototaxis (proportion of time spent in the dark side of the arena) and activity (number of times that the insect crossed the middle line of the arena). All assays were performed during the three first hours of scotophase, period in which triatomines normally exhibit an activity peak related to food search [16].

### Locomotory activity

**Fig 1:**
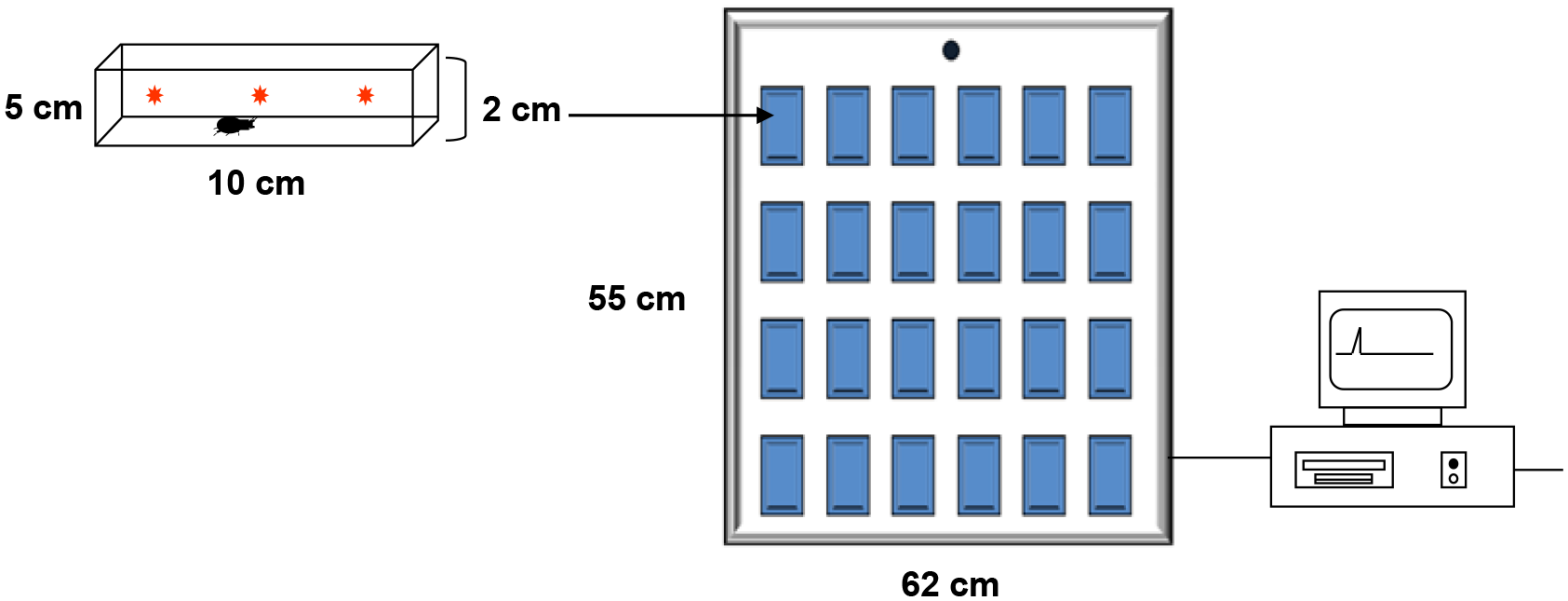
Schematic representation of the actometric set up. Each blue rectangle represents an actometric unit (10×5×2cm) arranged over a metal plate. Each unit presented three light barriers, indicated by red asterisks, distributed homogenously in the longitudinal direction in order to detect insect movement. The whole apparatus contained 40 units in total. Transparent acrylic arenas that fit tightly into the individual units were closed with a transparent lid to contain the insects during the assays. Insect movements interrupted the light beams generating signals recorded by a personal computer by means of an *ad hoc* software.

In order to record the locomotory activity of triatomine bugs, we developed an automatic actometer system (Fig 1) which was set up inside a controlled environment chamber (25±°C, 50±5%RH, photoperiod of 12:12 L/D). This device consisted of 40 individual arenas (10x5x2cm) arranged on an aluminum plate, each one presenting three light barriers, each constituted by a light emitting diode (LED) and a phototransistor (Fig 1). The activity of each insect was restricted to the interior of an acrylic container that acted as arena. During a trial, every time a moving insect interrupted a light beam, a signal was generated and recorded by an *ad hoc* software. Therefore, each of these signals was considered a locomotory activity event. During the photophase, fluorescent tubes located overhead illuminated the chamber at a light intensity of ca. 60 LUX. For the measurement of locomotory activity, 36 nymphs (control, n = 18; infected, n = 18) were placed individually in the arenas. Each container had filter paper as substrate and was covered with a rectangular acrylic lid. Insects were maintained in these conditions for 6 days, during which their activity was continuously recorded. This procedure was replicated three times for *T*. *cruzi* (uninfected, n = 54; infected, n = 54) and six times for *T*. *rangeli* (uninfected, n = 108; infected, n = 108) infection experiments.

### Identification and sequencing of the *R*. *prolixus foraging* gene

#### Gene identification and primer design

**Fig 2:**
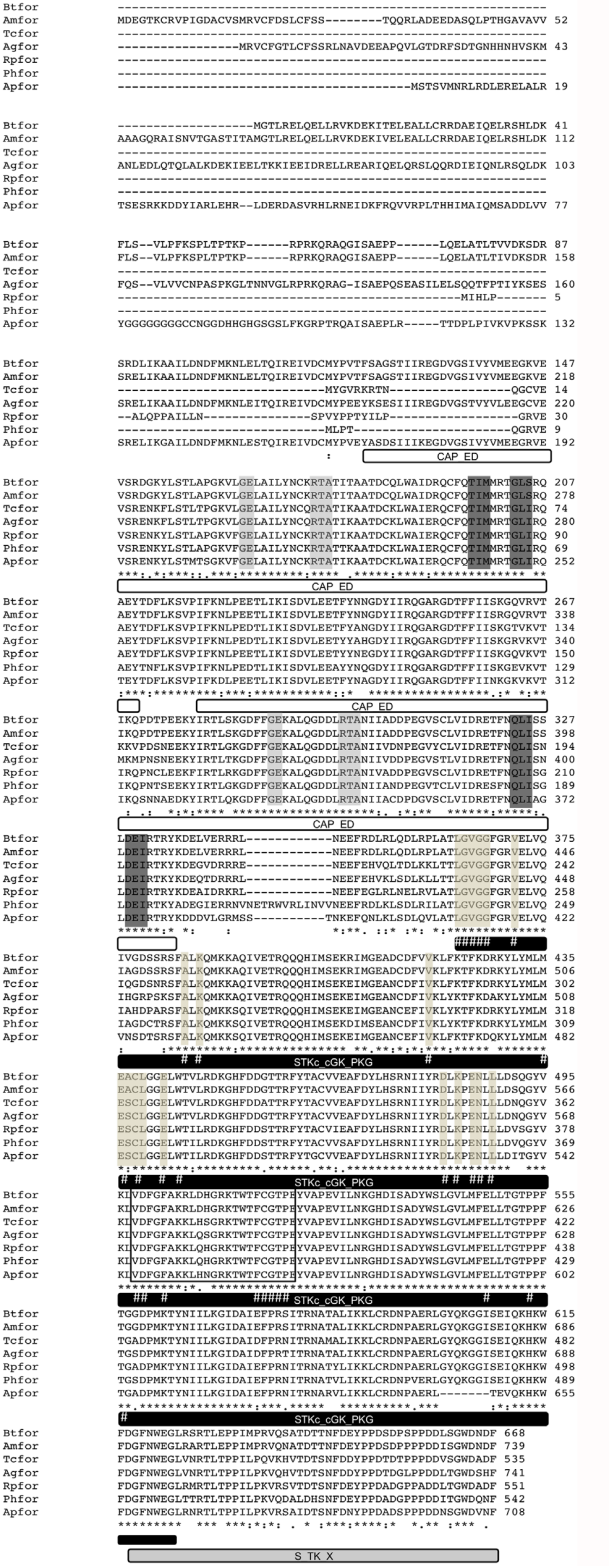
Amino acid sequence of *R*. *prolixus foraging* (*Rpfor*) protein. The putative coding sequence of the *R*. *prolixus foraging* gene is shown in comparison with orthologues from other insect species showing a high degree of conservation. Asterisks indicate identical amino acids, double points represent conserved exchanges and single points indicate homologous amino acids. The predicted protein for the *Rpfor* gene (code: RPRC000321-PA) contains 551 amino acids (1,656 bp) and a total of fourteen exons. The sequenced amplicon (from the position 195 to 1597) was identical to the *Rpfor* predicted protein sequence. The four conserved domains identified in the *Rpfor* gene are shown: two CAP-ED domains (effector domain of the CAP family of transcription factors), one STKc_cGK_PKG domain (catalytic domain of the cGMP- dependent protein kinase) and one S_TK_X domain (extension to Ser/Thr-type protein kinases). The second CAP-ED domain, the ATP binding sites and the active sites of the PKG catalytic domain are conserved between the species used in our analyses. Interestingly, the activation loop of the PKG catalytic domain presents three variable amino acid positions (378, 380 and 381). The first one presents a conserved amino acid change between arginine and lysine, both with polar and positively charged side chains. In the second position, aspartic acid, histidine and glutamine are present. The three amino acids are polar and present charged side chains: positive for histidine and glutamine, and negative for aspartic acid. Finally, the third position is the most variable considering amino acids properties and includes the polar amino acids: asparagine, histidine and serine. The light gray and the dark gray boxes indicate the ligand binding sites and the flexible hinges of the CAP-ED domains, respectively. The light brown boxes indicate the active sites of the STKc_cGK_PKG domain. The ATP binding sites are marked with #. The black box indicates the activation loop region. Sequence features were annotated manually using the *Rpfor* sequence as a reference. The *Rpfor* protein sequence has a similar length to those from *T*. *castaneum* and *P*. *humanus* with 535 and 542 amino acids, respectively. *B*. *terrestris*, *A*. *mellifera*, *An*. *glabripennis* and *Ac*. *pisum* present longer N-terminal regions (S1 Text).

The *foraging* gene (*Rpfor)* was identified in the *R*. *prolixus* genome (available in www.vectorbase.org) using tBLASTn v2.2.30 search [33] with orthologous sequences from UniProtKB/TrEMBL Database [34] of the following insect species: *Apis mellifera* (*Amfor*); *D*. *melanogaster* and *Pediculus humanus* (*Phfor*). The protein kinase (PF00069) and cyclic nucleotide-binding (PF00027) domains were identified in the *Rpfor* predicted protein sequence using *Pfam* v.27.0 [35]. Two pairs of primers were designed using the Primer3 4.0.0 software [http://primer3.ut.ee/] [36] to sequence a 1529 base pair (bp) fragment of the *Rpfor* coding sequence. The primers are shown in Table 1. Once the *Rpfor* was identified and sequenced, a multiple sequence alignment using Clustal X [37] was performed with orthologous sequences from other insects (Fig 2; *Acyrthosiphon pisum* (*Apfor*;) *Anoplophora glabripennis* (*Agfor*); *A*. *mellifera*; *Bombus terrestris* (*Btfor*); *Tribolium castaneum* (*Tcfor*) and *P*. *humanus*.

**Table 1:** Primer sequences, amplicon and intron lengths, squared correlation coefficient and qPCR efficiency.

Gene	Primer sequence (5'to 3')	Amplicon length (bp)	Intron length (bp)	R^2^	Eand
*Sequencing primers*					
*for-fragment 1*	For- AGGTGTTCGGAGAGTTGGCGATA	1100	-	-	-
	Rev- GCTACATACTCCGGCGTACCAC				
*for-fragment 2*	For- CCTGTTACTGGACGTTAGCGGTTA	429	-	-	-
	Rev- AAATCCGCATCCCAACCAGTGA				
*qPCR primers*					
*foraging*	For- AGTTCGAAGGTCTGCGACTG	191	3064	0.99	1.1
	Rev- GCCCATGATCTCCTTCTCGG				
β-*Actin*	For- TGTCTCCCACACTGTACCCATCTA	87	338	1	0.97
	Rev- TCGGTAAGATCACGACCAGCCAA				
*GAPDH*	For- GACTGGCATGGCATTCAGAGTT	182	1130	0.99	0.91
	Rev- CCCCATTAAAGTCCGATGACACC				

#### RNA extraction, cDNA synthesis and sequencing

Total RNA was extracted with TRIzol Reagent (Invitrogen, Carlsbad, CA, US) according to the manufacturer’s instructions from a pool of five brains and associated head muscles from infected and control insects. Total RNA concentrations were determined using a Qubit 2.0 Fluorometer (Life Technologies, Carlsbad, CA, US). DNAse treatment was performed with RQ1 RNase-Free DNases (Promega, Fitchburg, WI, US). Subsequently, all treated RNA (300 ng) was used to synthesize cDNA using SuperScript III Reverse Transcriptase (Life Technologies, Carlsbad, CA, US) and a mix 1:1 of Random Hexamers and Oligo(dT)_20_ primers in a final volume of 20μl. The cDNAs were used as templates for PCR reactions of the *Rpfor* gene which were performed for 35 cycles (94°C for 30s, 60°C for 30s and 72°C for 30s) with 1μl of cDNA, 200 μM of dNTPs, 200 nM of each primer and 1U of Taq polymerase (Promega, Fitchburg, WI, US) in a final volume of 20μl. PCR products were visualized on 2% agarose gels and purified using Wizard Genomic DNA Purification Kit (Promega, Fitchburg, WI, US). The sequencing reactions for the purified product were performed with both primers using an ABI Prism BigDye V 3.1 Terminator Cycle Sequencing kit and an ABI 3730 DNA sequencing system (Life Technologies, Carlsbad, CA, US). The consensus sequences were obtained using the Staden Package 2.0 [38] and verified by comparison with the *R*. *prolixus* genomic database, using the nucleotide basic local alignment search tool (BLASTn).

### Quantitation of *Rpfor* gene expression

Brains and pieces of surrounding muscles were dissected on a freeze cold dissecting dish (BioQuip, Gardena, CA, US) and remained frozen during the entire dissection procedure. Samples consisted of pools of these tissues dissected from five 5^th^ instar nymphs (n = 3 biological replicates for each treatment, except for insects used as controls for *T*. *rangeli* infection, n = 2). RNA was extracted on the same day in which dissection happened using TRI Reagent (Sigma-Aldrich, St. Louis, MO, US) according to the manufacturer’s instructions. RNA concentrations were determined using a BIOPhotometer (Eppendorf, Hamburg, Germany). Total RNA (500ng) was used for reverse transcription using M-MLV Reverse Transcriptase (Promega, Fitchburg, WI, US) and a modified oligo dT primer (MgdT 5’-CGGGCAGTGAGCAACG (T12)-3’) as described [39]. Quantitative PCR (qPCR) was used to assess whether *Rpfor* expression levels were affected by trypanosome infection. All reactions contained 1μl of cDNA, 5ng/μl of each primer and 6μl of PerfeCTa SYBR Green Super Mix (Quanta Biosciences, Gaithersburg, MD, US) in a final volume of 10μl. The reactions were conducted in a RotorGene 3000 thermal cycler (Corbett Research, Sydney, Australia). The qPCR conditions used were: 95°C: 2min, 35 cycles of 95°C: 10s, 60°C(*Rpfor*), 55°C(β-actin and GADPH): 10s and 72°C: 30s, followed by a melting curve analysis to confirm the specificity of the reaction. In all qPCR experiments, no-template controls (NTC) were included for each primer set to verify the absence of exogenous DNA and primer-dimers. Reactions on each sample were run in duplicate. Relative differences in abundance of *Rpfor* transcripts were calculated using the 2–ΔΔCt method [40] with β-actin as reference gene as described [41]. The PCR efficiencies (E) and repeatability (R^2^) for each primer were determined using the slope of a linear regression model (Table 1) [42]. All data were normalized relative to values recorded for control insects.

### Statistical analysis

Statistical analyses were performed using R 3.0.2 [43]. Negative phototaxis (i.e. proportion of time spent in the dark side of the arena) in uninfected *vs*. infected insects was analyzed using linear regression with beta distribution and logit link function (function *betareg* in betareg package) [44], since values could only range from 0 to 1. To compare the activity (i.e. number of times insects crossed the middle line) in the same experiment, generalized linear regression with Poisson error distribution and log link function was used.

The locomotory activity of *R*. *prolixus* individuals in both experiments (i.e. effects of *T*. *cruzi* and *T*. *rangeli*) was analyzed with linear mixed-effects models (function *lmer* in lme4 package) [45] fitted by restricted maximum likelihood (REML). Square root transformed locomotory activity data were used as a response variable whereas treatment (uninfected *vs*. infected) and time of the day (24 hour period) were set as fixed variables. To take into account that the data were measured repeatedly on the same individuals with time intervals of one hour, individuals were included as random factor. Likewise, time of the day was also measured repeatedly over the experiment and it was hence included as random factor nested within the variable day. Pairwise contrasts (function *testInteractions* in phia package) [46] were used to evaluate the locomotory activity of uninfected and infected individuals at every hour of the day. P-values of the contrasts were adjusted by Holm-Bonferroni method to correct for the problem of multiple comparisons.

### Accession numbers

*D*. *melanogaster* (Q03043); *Apfor* (H6V8U7); *Agfor* (V5GSC1); *Amfor* (T1SGQ4); *Btfor* (C6GBY7); *Tcfor* (D6WXB3); *Phfor* (E0VGN7).

## Results

### *T*. *rangeli* infection affects negative phototaxis of *R*. *prolixus*

**Fig 3:**
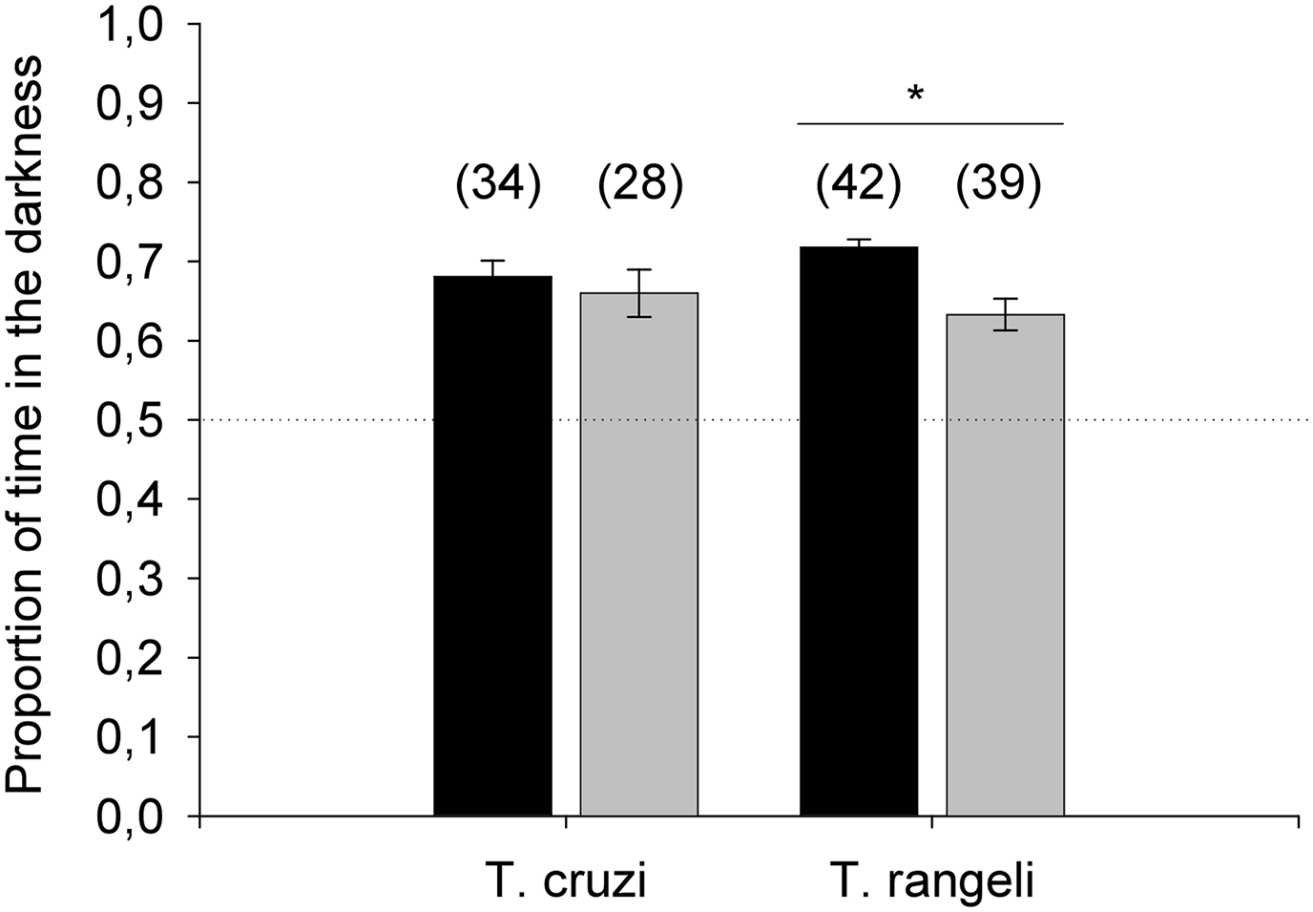
*Rhodnius prolixus* nymphs infected by *Trypanosoma rangeli* show altered phototaxis. Time spent in the dark and light sides of an arena was recorded for all infected and uninfected insects for 10 min. The proportion of time spent in the dark side was used as a measure of negative phototaxis. Black and gray bars represent the mean ± SE of the proportion of time spent in dark area for control and infected individuals, respectively. Numbers in parentheses indicate the number of insects tested in each experiment. The dotted line at 0.5 indicates a reference for lack of preference between the light or dark halves of the arena. *p = 0.01.

Triatomine insects exhibit a strong aversion to light as an adaptive aspect of their behavior. As trypanosome infection has been shown to affect several parameters of the biology of their invertebrate vectors, we decided to investigate whether these parasites could influence triatomine negative phototaxis. By analyzing the amount of time that uninfected controls, as well as *T*. *cruzi* or *T*. *rangeli-*infected insects, spent on either the dark or the light sides of a chamber we evaluated this behavior. During the assays, uninfected and *T*. *cruzi*-infected insects crossed the middle line between the two sectors of the arena on average 14 and 13 times, respectively (Z = 55.77, p = 0.39). Uninfected *R*. *prolixus* spent more than 70% of the time on the dark side of the arena and *T*. *cruzi*-infected insects showed a similar behavior (Fig 3; Z = 4.74, p = 0.92). Likewise, our experiments with *T*. *rangeli*-infected insects showed that the number of crossings between the two sections of the arena was not significantly different between uninfected or *T*. *rangeli-*infected triatomines (12 and 11 times respectively on average, Z = 55.36, p = 0.12). However, the percentage of time spent in the dark sector was significantly reduced for *T*. *rangeli*-infected insects compared to uninfected controls (Fig 3; Z = 6.83, p = 0.01).

### Trypanosome infection modifies locomotory activity of *R*. *prolixus*

**Fig 4:**
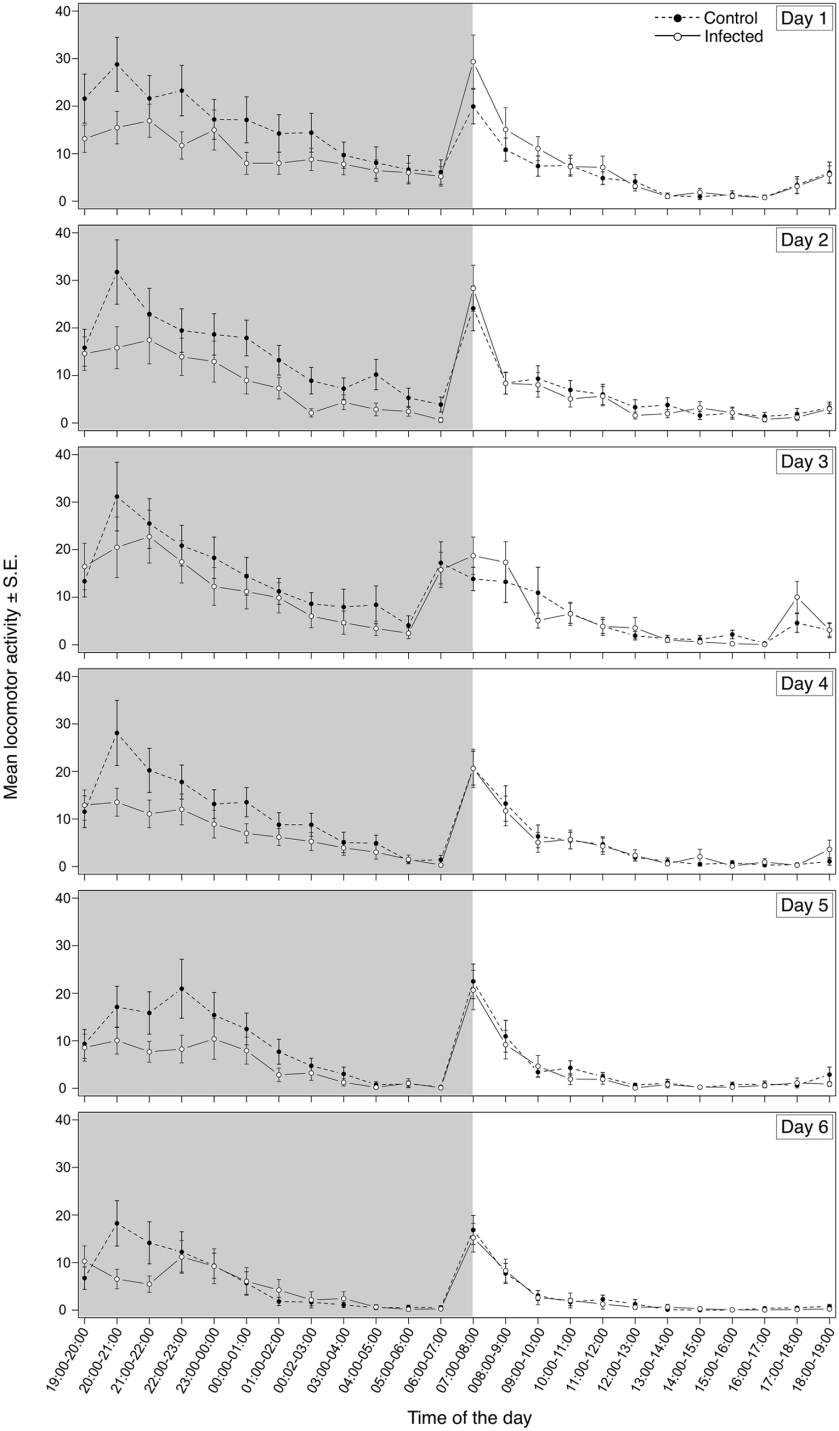
*Trypanosoma cruzi* infection decreases locomotion of *Rhodnius prolixus* during scotophase. *T*. *cruzi-*infected and uninfected *R*. *prolixus* nymphs were individually placed inside actometer units and their movements recorded for 6 days. White and grey areas depict the photophase and scotophase, respectively. Data are presented as the square root of the mean locomotory activity of three replicate runs (n = 54 insects for each treatment).

In addition to evaluating the effect of trypanosome infection on triatomine phototaxis we tested whether locomotion could also be affected. *R*. *prolixus* exhibit a bimodal pattern of daily locomotion activity and we used an actometer to evaluate if infection by *T*. *cruzi* or *T*. *rangeli* could interfere with this aspect of triatomine behavior. The movements of uninfected and infected insects were monitored in order to detect variations in their locomotory activity. Uninfected and *T*. *cruzi*-infected insects showed a similar pattern of locomotion consisting of two main peaks: one at the second hour of the scotophase and the other, during the first hour of the photophase. During the remaining time, especially during the photophase, insects exhibited almost no locomotion. However, we found a statistically significant interaction between infection status and hour of the daily cycle in insects infected by *T*. *cruzi* (Fig 4; F = 4.02, p<0.0001). Pairwise contrasts revealed that *T*. *cruzi* infected insects showed significantly decreased locomotory activity during the second hour of the scotophase (20:00–21:00h; S1 Table). In overall, the total number of movements recorded for *T*. *cruzi*-infected insects was about 20% less than that observed in uninfected control insects.

**Fig 5:**
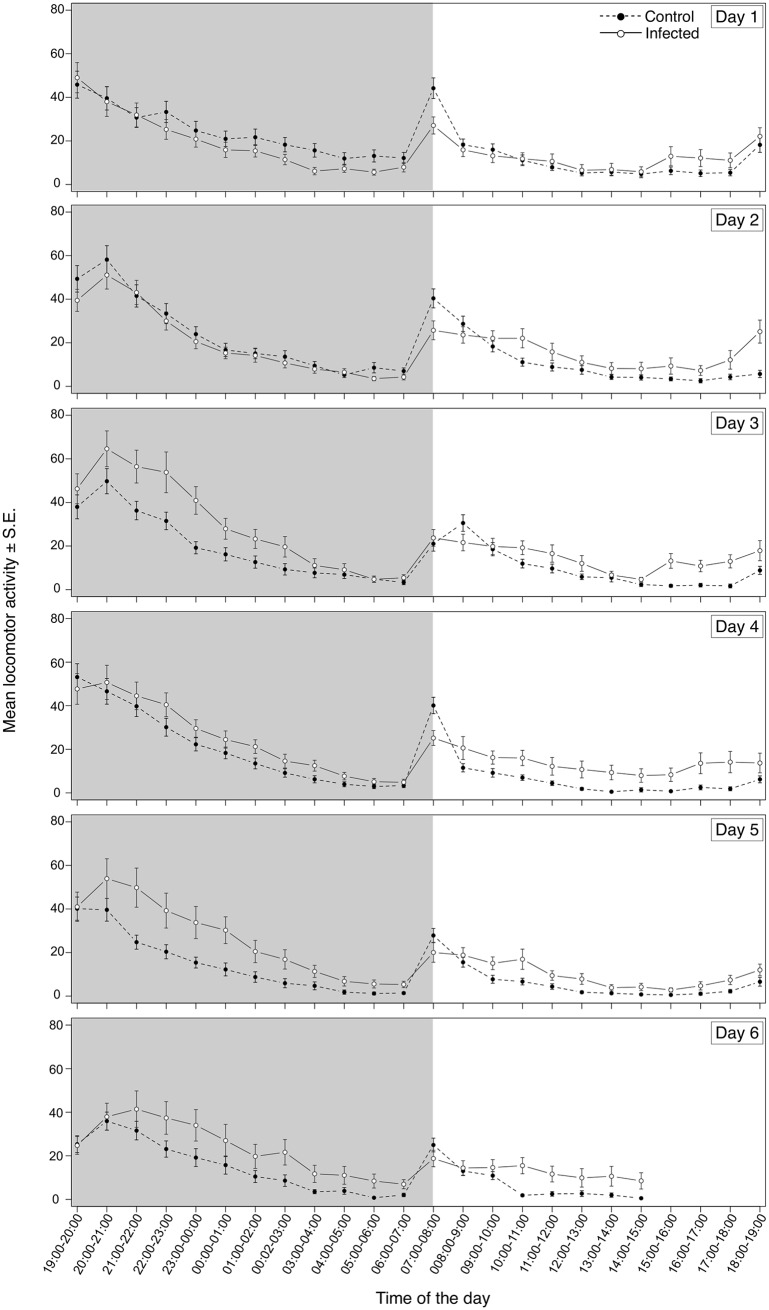
*Trypanosoma rangeli* infection increases locomotion of *Rhodnius prolixus*. *T*. *rangeli-*infected and uninfected *R*. *prolixus* nymphs were individually placed inside actometer units and their movements were recorded for 6 days. White and grey areas depict the photophase and scotophase, respectively. Data are presented as the square root of the mean locomotory activity of six replicate runs (n = 108 insects for each treatment).

When *T*. *rangeli*-infected insects were analyzed, a similar general pattern of daily activity with two peaks was observed. As seen for the *T*. *cruzi* experiment, infection by *T*. *rangeli* also promoted alterations in *R*. *prolixus* motility at a particular time of the day (Fig 5; F = 8.70, p<0.0001). However, differently from the former results, *T*. *rangeli*-infected nymphs showed an increase in their general activity that was significant during the 11^th^ and 12^th^ hours of the photophase (17:00–18:00, 18:00–19:00h; S2 Table) when compared to uninfected controls. The total number of movements displayed by *T*. *rangeli*-infected insects was on average 23% higher than that shown by uninfected control insects.

### Trypanosome infection interferes with the expression of *Rpfor* in *R*. *prolixus*

Many aspects of vector behavior are controlled by complex and yet unknown genetic interactions and signaling pathways. Since parasites can interfere with gene expression from their invertebrate vectors, we speculated whether the levels of the *foraging* gene (shown to modulate locomotion in other species) would be altered in trypanosome-infected *R*. *prolixus*. We identified an ortholog of the *foraging gene (Rpfor)* in the supercontig GL553754, between the positions 4,659 and 26,665 on the forward strand, in the *R*. *prolixus* genome database. The predicted protein for *Rpfor* gene (code: RPRC000321-PA) contains 551 amino acids (1,656 bp) is coded by fourteen exons and is similar in length to those from *T*. *castaneum* and *P*. *humanus* with 535 and 542 amino acids, respectively. Furthermore, the presence of functional domains characteristic of this gene was confirmed in the *Rpfor* protein sequence (Fig 2).

**Fig 6:**
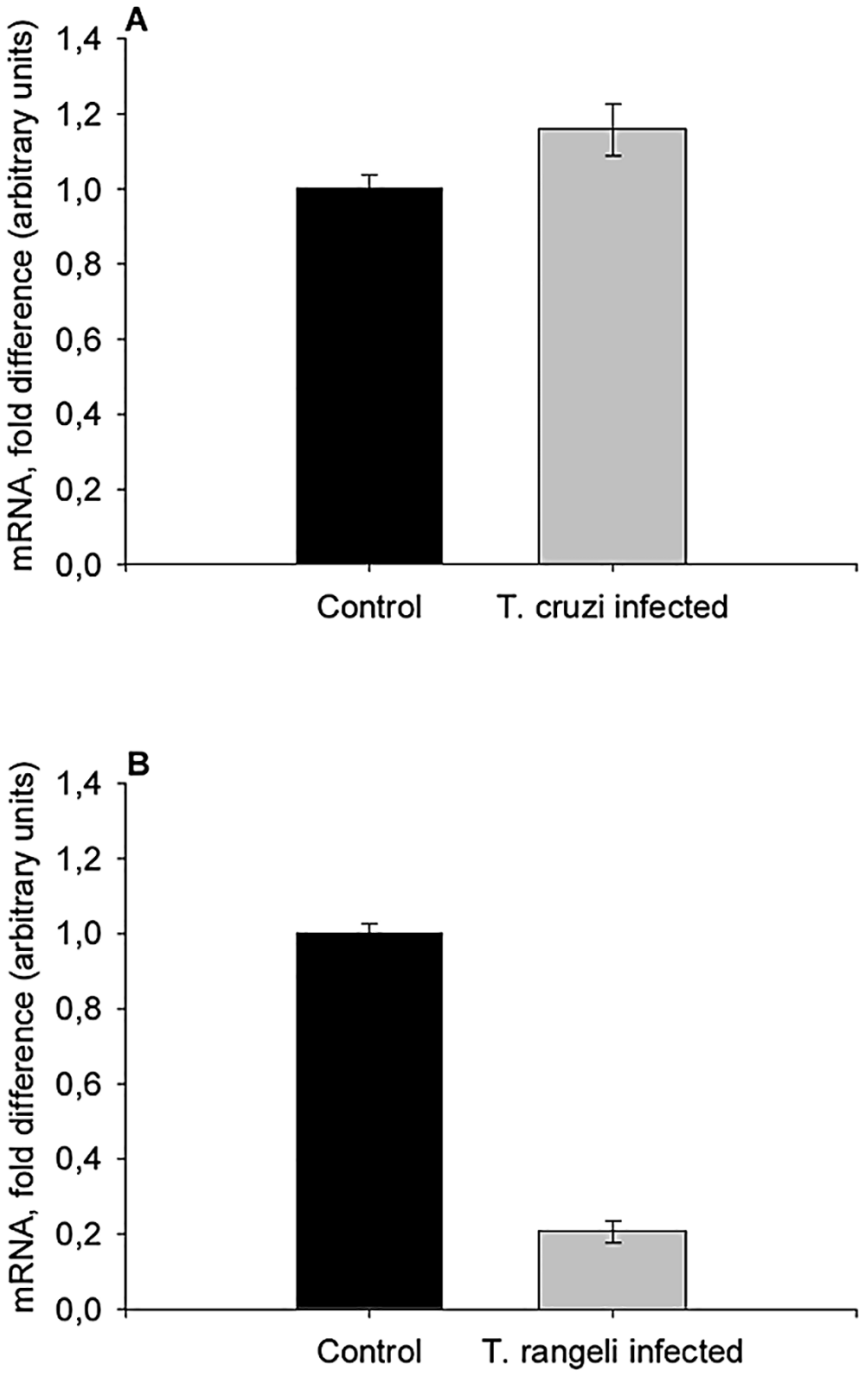
*Foraging* gene expression is decreased in *R*. *prolixus* infected with *T*. *rangeli*. Expression of *Rpfor* was evaluated by qPCR in uninfected and (A) *T*. *cruzi-* or (B) *T*. *rangeli-*infected *R*. *prolixus* nymphs. Bars represent the mean fold change of *Rpfor* mRNA levels normalized relative to those of uninfected control insects ± SE (converted to same arbitrary scale as the means); n = 3 pools (5 brains each) per treatment, except for *T*. *rangeli* controls, n = 2 pools (5 brains each).

Using qPCR analyses we quantified the relative expression of *Rpfor* in uninfected and trypanosome-infected *R*. *prolixus*. We found no difference in *Rpfor* expression levels between *T*. *cruzi* infected and uninfected insects (Fig 6A). In contrast, however, when the insects were infected with *T*. *rangeli*, there was an important decrease in *Rpfor* expression when compared with uninfected controls (Fig 6B). We observed a decrease of 4.8 fold in the relative expression levels of *Rpfor* in *T*. *rangeli* infected insects.

## Discussion

Triatomines are nocturnal insects and remain inside protected shelters during daylight hours [17]. Such behaviors are highly adaptive as they allow triatomines to decrease their exposure to predators, which eventually become hosts from whom these insects obtain their blood meals. In a natural context, a higher exposure during daylight hours could potentially increase insect mortality by predation. Uninfected and *T*. *cruzi*-infected nymphs of *R*. *prolixus* showed a marked negative phototaxis, spending more than 70% of the time in the dark sector of the arena. Interestingly, *T*. *rangeli*-infected triatomines seem to be less averse to light, and spent a significantly shorter proportion of time in the dark. Therefore, the decreased negative phototaxis of *T*. *rangeli*-infected insects shown in the present work could be an indication of a behavioral alteration potentially costly to the insects.

Independent of infection status, all *R*. *prolixus* nymphs presented a bimodal pattern of locomotory activity with pronounced peaks at the start of the scotophase and photophase, in agreement with patterns described for triatomines [15, 16]. Interestingly, *T*. *cruzi*-infected nymphs showed decreased spontaneous locomotory activity during the first half of the scotophase. This interval represents the period in which triatomines search for food and sexual partners [15, 16, 47]. Altered host activity is a common effect of parasitism [48]. In some cases, a declined activity may be caused by tissue destruction as seen in *A*. *aegypti* infected by *Brugia pahangi* [49] and in *Gryllus integer* infected by *Ormia ochracea* [50]. However, *T*. *cruzi* does not invade the celomic cavity of its vectors and is restricted to the intestinal tract. Triatomine tissue injuries have not been reported during *T*. *cruzi* infection [51], indicating that a decrease of activity as a consequence of direct damage is unlikely. Different effects on triatomine fitness as a result of *T*. *cruzi* infection have previously been described, including resistance to starvation [8], delayed molt and increased mortality [52]. Recently, our laboratory team showed that *T*. *cruzi* can be pathogenic to its vectors depending on the environmental temperature and insect nutritional status [10, 30]. Taken together, these data suggest that *T*. *cruzi* competes with its vector for nutrients, since starved infected insects have reduced survival and increased susceptibility to other stress factors [53]. Furthermore, *T*. *cruzi*-infected *T*. *infestans* need more blood for molting than uninfected controls, probably as a compensation for the nutrients lost to trypanosomes [7]. In addition, a reduction in gonad weight as a consequence of nutrition curtailment was observed in the triatomine *Mepraia spinolai* infected with *T*. *cruzi* [54]. As well, infected *M*. *spinolai* find hosts almost twice as fast as uninfected bugs [9]. In our experimental design, insect activity was evaluated in the absence of host cues. Therefore, it is possible that the decreased locomotory activity observed in *T*. *cruzi*-infected insects could represent an energy saving mechanism designed to avoid the loss of already lowered nutritional resources in the absence of host cues. This lack of activity when host stimuli are absent and no indication that a potential blood meal is available might be intended to preserve insect fitness. A similar protective behavior has been suggested for *C*. *lectularius* in the absence of host cues [20].

Contrarily to what was observed with *T*. *cruzi* infected bugs, infection of *R*. *prolixus* with *T*. *rangeli* promoted instead, an increased locomotory activity during most of the daily cycle. Exceptionally, *T*. *rangeli* infected insects consistently showed a decreased locomotory activity at the onset of light. It is worth noting that the lights-on period normally induces a certain increase in locomotion in animals as a consequence of such abrupt change in light intensity, independently of having a circadian component superimposed. In the case of bugs infected with this parasite, their decreased negative phototaxis could at least partially explain this altered pattern in comparison to control insects. Nevertheless, the results for the remaining of the photophase together with the decrease in negative phototaxis shown in our experiment suggest that *T*. *rangeli* promotes an increased exposure of insects to predators. Whether this alteration increases parasite transmission still needs to be tested. An increase in the locomotory activity of infected vectors has also been reported in *A*. *aegypti*/Dengue 2 [55] and *A*. *aegypti*/*Wolbachia pipientis* [56] associations. In both cases, increases in pathogen transmission rates have been suggested.

The origin and evolutionary distance of *T*. *cruzi* and *T*. *rangeli* is controversial, as a new perspective, the bat seeding hypothesis, proposes that *T*. *cruzi* evolved from bat trypanosomes [57]. According to this, *T*. *cruzi* would have evolved more recently than proposed in the prevailing Southern super-continent hypothesis [57]. In spite of this, *T*. *cruzi* and *T*. *rangeli* are classified unequivocally in the same clade [58, 59]. In case of an ancient *T*. *cruzi* origin, the divergence time between *T*. *cruzi* and *T*. *rangeli*, would have occurred several millions of years ago [60]. This scenario would have possibly promoted distinct evolutionary associations with their insect vectors which concur with the obvious lifecycle and morphological differences between them. For *T*. *rangeli*, it has been accepted that the parasite presents a close evolutionary association with *Rhodnius spp*. [61] which has promoted the appearance of parasite strains closely associated to specific *Rhodnius* species [62]. Whether co-evolution enabled these parasites to manipulate vector behavior to increase their own transmission is an intriguing question for future studies.

The molecular mechanisms underlying the modification of vector locomotion by parasites are still largely unknown, but likely require alterations in gene expression. Insect locomotory activity can be modulated regulating the expression of a gene coding for a PKG named *foraging* (see rev. [63, 64]). Therefore, we investigated whether *Rpfor* expression levels were altered in trypanosome-infected triatomines. *Rpfor* expression was differently affected in *R*. *prolixus* infected with *T*. *cruzi* and *T*. *rangeli*. While infection by *T*. *cruzi* promoted a trend for increased *Rpfor* mRNA abundance, *T*. *rangeli* infection, in contrast, significantly decreased *Rpfor* expression. The relation between locomotory activity, food search and the levels of *foraging* gene expression has been studied in different organisms (see rev. in [63, 64]). Foraging behavior is largely influenced by cGMP-activated protein kinase pathways across taxa, although the mechanisms involved still remain elusive. Two opposite models connecting insect locomotion/pattern of behavior and *foraging* gene expression have been described to date. In the first case, increased *for* expression has been related to increased locomotory activity in fruit flies [65], bees [66], bumblebees [67] and locusts [68]. In contrast, higher levels of *for* expression have been related to a decreased locomotory activity in ants [69], wasps [70] and nematodes [71]. Interestingly, our data from *T*. *rangeli*-infected insects suggest a relation fitting the second model. These data strongly support the need for future studies evaluating *Rpfor* activity in triatomines under different physiological conditions, as well as gene expression manipulation or modulation of PKG activity. To our knowledge, this is the first demonstration that both, the expression of the *for* gene and the behavior of an insect host vectoring human disease have been shown to be altered by a parasite.

It is relevant to consider that potential limitations may affect the conclusions of this study according to the methods used to test our hypotheses. One first case would be represented by the fact that we suggest that infection by *T*. *rangeli* induces a decrease in bug negative phototaxis. While we have followed the methodology used by Reisenman and colleagues [72] and Reisenman and Lazzari [32] because we consider those reports to be very relevant in relation to the existing literature about triatomine behavior, one may argue that these laboratory conditions can have restricted predictor power for natural environments. We highlight here that the size of an arena and the duration of an experiment only serve the purpose of evincing an effect hypothesized previously. If such effects can be put in evidence using those conditions, then they seem adequate. In fact, our results seem to indicate that our methods were adequate to prove that *T*. *rangeli* infection affects this parameter. The relative weight of such an effect in a natural scenario is not evident from them, and should be the focus of future studies. A similar case applies to the study of the locomotory activity of *R*. *prolixus*. Our arenas seemed effective for measuring bug locomotory activity and evince alterations. Furthermore, the scale of arena used was similar to those traditionally used for actometer studies with diverse insects [16, 73, 74]. Another issue that could potentially be raised would be that of the infection procedures used. In the case of *T*. *rangeli*, we have injected a small inoculum, based on our previous study evaluating the effects of different amounts of *T*. *rangeli* on *R*. *prolixus* nymphs [14]. For *T*. *cruzi* infections, we used a relatively large inoculum based on the fact that there is a relevant reduction in *T*. *cruzi* populations after the initial five days of infection [75]. It is worth mentioning that natural infections in sylvatic cycles probably include quite different conditions represented by diverse mammals experiencing acute or chronic infections. This includes opossums, which are known to show very high parasitemia [76]. It is difficult for us to determine an adequate standard for this, but we tend to assume that our conditions are quite reasonable. Finally, since our intent was to test whether parasites alter locomotory patterns in *R*. *prolixus*, the use of nymphs was planned to exclude the potential interference of sexual activities that could have interfered if adult bugs were used. Confirming an effect of trypanosome infection on 5^th^ instar nymphs supports our claim. It is worth mentioning that a recently published report has shown that adult reproduction is compromised by infection with either of these parasites [10], suggesting that adults may suffer similar consequences of trypanosome infection.

This report proposes a new approach in the study of trypanosome-triatomine interactions, showing that these parasites alter bug locomotory activity and, in the case of *T*. *rangeli*, the phototatic behavior and the expression of a gene that has been shown to modulate insect behavior. Taken together, these alterations would possibly affect parasite transmission rates. Interestingly, it has recently been shown that *T*. *cruzi* induces alterations in the dispersion of *Triatoma dimidiata* females [77], as well as in the wing size of adults of this species [78]. In addition, *T*. *rangeli* promotes longer flights in *Rhodnius pallescens*, possibly affecting its dispersion ability [79]. Altogether, these facts evidence that triatomine locomotion and trypanosome infection seem to be connected but the mechanisms through which these effects take place remain obscure. We suggest that functional genomics studies should enable a better understanding of the molecular mechanisms underlying the trypanosome induced alterations of triatomine behavior.

## Supporting Information

S1 Text Complete foraging protein sequences from *R*. *prolixus* and other insect species. (DOCX)

S1 Table Pairwise contrasts of the linear mixed-effects model between *T*. *cruzi*-infected and uninfected insects. Pairwise contrasts were used to evaluate the locomotory activity of uninfected and infected individuals at every hour of the day. P-values of the contrasts were adjusted by Holm-Bonferroni method to correct for the problem of multiple comparisons.
(DOCX)

S2 Table Pairwise contrasts of the linear mixed-effects model between *T*. *rangeli*-infected and uninfected insects. Pairwise contrasts were used to evaluate the locomotory activity of uninfected and infected individuals at every hour of the day. P-values of the contrasts were adjusted by Holm-Bonferroni method to correct for the problem of multiple comparisons.
(DOCX)
