# Supplementary material for: Trypanosomes Modify the Behavior of Their Insect Hosts: Effects on Locomotion and on the Expression of a Related Gene
Source: PLoS Negl Trop Dis. 2015 Aug 20;9(8):e0003973. doi: 10.1371/journal.pntd.0003973 (PMC4546274; doi:10.1371/journal.pntd.0003973)
Supplement: S1 Text — (DOCX) [file pntd.0003973.s001.docx]

Complete foraging protein sequences from *R. prolixus* and other insect species used in Figure 2.

>Rpfor

MIHLPALQPPAILLNSPVYPPTYILPGRVEVSRENKYLSTLAPGKVFGELAILYNCKRTA

TIKAATDCKLWAIERQCFQTIMMRTGLIRQAEYTDFLKSVPIFKNLPEETLIKISDVLEE

TYYNEGDYIIRQGARGDTFFIISKGQVKVTIRQPNCLEEKFIRTLRKGDFFGEKALQGDD

LRTANIVADDPEGVSCLVIDRETFNQLISGLDEIRTRYKDEAIDRKRLNEEFEGLRLNEL

RVLATLGVGGFGRVELVQIAHDPARSFALKQMKKSQIVETRQQQHIMSEKEIMGEANCEF

IVKLYKTFKDRKYLYMLMESCLGGELWTILRDKGHFDDSTTRFYTACVIEAFDYLHSRNI

IYRDLKPENLLLDVSGYVKLVDFGFAKKLQHGRKTWTFCGTPEYVAPEVILNRGHDISAD

YWSLGVLMFELLTGTPPFTGADPMKTYNIILKGIDAIEFPRNITRNATYLIKKLCRDNPA

ERLGYQKGGISEIQKHKWFDGFNWEGLRMRTLTPPILPKVRSVTDTSNFDEYPPDADGPP

ADDLTGWDADF

>Apfor

MSTSVMNRLRDLERELALRTSESRKKDDYIARLEHRLDERDASVRHLRNEIDKFRQVVRP

LTHHIMAIQMSADDLVVYGGGGGGGGCCNGGDHHGHGSGSLFKGRPTRQAISAEPLRTTD

PLPIVKVPKSSKSRELIKGAILDNDFMKNLESTQIREIVDCMYPVEYASDSIIIKEGDVG

SIVYVMEEGRVEVSRENKYLSTMTSGKVFGELAILYNCKRTATIKAATDCKLWAIERQCF

QTIMMRTGLIRQTEYTDFLKSVPIFKDLPEETLIKISDVLEETFYNAGDYIIRQGARGDT

FFIINKGKVKVTIKQSNNAEDKYIRTLQKGDFFGEKALQGDDLRTANIIACDPDGVSCLV

IDRETFNQLIAGLDEIRTRYKDDDVLGRMSSTNKEFQNLKLSDLQVLATLGVGGFGRVEL

VQVNSDTSRSFALKQMKKSQIVETRQQQHIMSEKEIMGEANCEFIVKLFKTFKDQKYLYM

LMESCLGGELWTILRDKGHFDDSTTRFYTGCVVEAFDYLHSRNIIYRDLKPENLLLDITG

YVKLVDFGFAKKLHNGRKTWTFCGTPEYVAPEVILNRGHDISADYWSLGVLMFELLTGTP

PFTGADPMKTYNIILKGIDAIEFPRNITRNARVLIKKLCRDNPAERLTEVQKHKWFDGFN

WEGLRNRTLTPPILPKVRSAIDTSNFDNYPPDADSPPPDDNSGWDVNF

>Tcfor

MYGVRKRTNQGCVEVSRENKFLSTLTPGKVLGELAILYNCQRTATIKAATDCKLWAIERQ

CFQTIMMRTGLIRQAEYTDFLKSVPIFKNLPEDTLIKISDVLEETFYANGDYIIRQGARG

DTFFIISKGTVKVTKKVPDSNEEKYIRTLGKGDFFGEKALQGDDLRTANIIVDNPEGVYC

LVIDRETFNQLISNLDEIRTKYKDEGVDRRRENEEFEHVQLTDLKKLTTLGVGGFGRVEL

VQIQGDSNRSFALKQMKKAQIVETRQQQHIMSEKEIMGEANCDFIVKLFKTFKDRKYLYM

LMESCLGGELWTVLRDKGHFDDATTRFYTACVVEAFDYLHSRNIIYRDLKPENLLLDNQG

YVKLVDFGFAKKLHSGRKTWTFCGTPEYVAPEVILNKGHDISADYWSLGVLMFELLTGTP

PFTGADPMKTYNIILKGIDAIDFPRNITRNAMALIKKLCRDNPAERLGYQKGGISEIQKH

KWFDGFNWEGLVNRTLTPPILPQVKHVTDTSNFDDYPPDTDTPPPDDVSGWDADF

>Phfor

MLPTQGRVEVSRENKYLSTLAPGKVFGELAILYNCKRTATTKAATDCKLWAIERQCFQTI

MMRTGLIRQAEYTNFLKSVPIFKNLPEDTLIKISDVLEEAYYNQGDYIIRQGARGDTFFI

ISKGEVKVTIKQPNTSEEKYIRTLGKGDFFGEKALQGDDLRTANIIANDPEGVTCLVIDR

ESFNQLISGLDEIRTKYADEGIERRNVNETRWVRLINVVNEEFRDLKLSDLRILATLGVG

GFGRVELVQIAGDCTRSFALKQMKKSQIVETRQQQHIMSEKDIMSEANCDFIVKLYKTFK

DRKYLYMLMESCLGGELWTILRDKGHFDDSTTRFYTACVVSAFDYLHSRNIIYRDLKPEN

LLLDVQGYVKLVDFGFAKKLQHGRKTWTFCGTPEYVAPEVILNRGHDISADYWSLGVLMF

ELLTGTPPFTGSDPMKTYNIILKGIDAIEFPRNITRNATVLIKKLCRDNPVERLGYQKGG

ISEIQKHKWFDGFNWEGLTTRTLTPPILPKVQDALDHSNFDEYPPDADGPPPDDITGWDQ

NF

>Btfor

MGTLRELQELLRVKDEKITELEALLCRRDAEIQELRSHLDKFLSVLPFKSPLTPTKPRPR

KQRAQGISAEPPLQELATLTVVDKSDRSRDLIKAAILDNDFMKNLELTQIREIVDCMYPV

TFSAGSTIIREGDVGSIVYVMEEGKVEVSRDGKYLSTLAPGKVLGELAILYNCKRTATIT

AATDCQLWAIDRQCFQTIMMRTGLSRQAEYTDFLKSVPIFKNLPEETLIKISDVLEETFY

NNGDYIIRQGARGDTFFIISKGQVRVTIKQPDTPEEKYIRTLSKGDFFGEKALQGDDLRT

ANIIADDPEGVSCLVIDRETFNQLISSLDEIRTRYKDELVERRRLNEEFRDLRLQDLRPL

ATLGVGGFGRVELVQIVGDSSRSFALKQMKKAQIVETRQQQHIMSEKRIMGEADCDFVVK

LFKTFKDRKYLYMLMEACLGGELWTVLRDKGHFDDGTTRFYTACVVEAFDYLHSRNIIYR

DLKPENLLLDSQGYVKLVDFGFAKRLDHGRKTWTFCGTPEYVAPEVILNKGHDISADYWS

LGVLMFELLTGTPPFTGGDPMKTYNIILKGIDAIEFPRSITRNATALIKKLCRDNPAERL

GYQKGGISEIQKHKWFDGFNWEGLRSRTLEPPIMPRVQSATDTTNFDEYPPDSDPSPPDD

LSGWDNDF

>Agfor

MRVCFGTLCFSSRLNAVDEEAPQVLGTDRFSDTGNHHNHVSKMANLEDLQTQLALKDEKI

EELTKKIEEIDRELLREARIQELQRSLQQRDIEIQNLRSQLDKFQSVLVVCNPASPKGLT

NNVGLRPRKQRAGISAEPQSEASILELSQQTFPTIYKSESSRELIKAAILDNDFMKNLEI

TQIREIVDCMYPEEYKSESIIIREGDVGSTVYVLEEGCVEVSRENKFLSTLTPGKVLGEL

AILYNCQRTATIKAATDCKLWAIERQCFQTIMMRTGLIRQAEYTDFLKSVPIFKNLPEDT

LIKISDVLEETYYAHGDYIIRQGARGDTFFIISKGRVKVTMKMPNSNEEKYIRTLTKGDF

FGEKALQGDDLRTANIIVDDPEGVSTLVIDRETFNQLISNLDEIRTKYKDEQTDRRRLNE

EFEHVKLSDLKLLTTLGVGGFGRVELVQIHGRPSKSFALKQMKKAQIVETRQQQHIMSEK

EIMGEANCDFIVKLYKTFKDAKYLYMLMESCLGGELWTVLRDKGHFDDGTTRFYTACVVE

AFDYLHSRNIIYRDLKPENLLLDNQGYVKLVDFGFAKKLQSGRKTWTFCGTPEYVAPEVI

LNKGHDISADYWSLGVLMFELLTGTPPFTGSDPMKTYNIILKGIDAIDFPRTITRNATAL

IKKLCRDNPAERLGYQKGGISEIQKHKWFDGFNWEGLVNRTLTPPILPKVQHVTDTSNFD

DYPPDTDGLPPDDLTGWDSHF

>Amfor

MDEGTKCRVPIGDACVSMRVCFDSLCFSSTQQRLADEEDASQLPTHGAVAVVAAAGQRAI

SNVTGASTITAMGTLRELQELLRVKDEKIVELEALLCRRDAEIQELRSHLDKFLSVLPFK

SPLTPTKPRPRKQRAQGISAEPPLQELATLTIVDKSDRSRELIKAAILDNDFMKNLELTQ

IREIVDCMYPVTFSAGSTIIREGDVGSIVYVMEEGKVEVSRDGKYLSTLAPGKVLGELAI

LYNCKRTATITAATDCQLWAIDRQCFQTIMMRTGLSRQAEYTDFLKSVPIFKNLPEETLI

KISDVLEETFYNNGDYIIRQGARGDTFFIISRGQVRVTIKQPDTPEEKYIRTLSKGDFFG

EKALQGDDLRTANIIADDPEGVSCLVIDRETFNQLISSLDEIRTRYKDELVERRRLNEEF

RDLRLQDLRPLATLGVGGFGRVELVQIAGDSSRSFALKQMKKAQIVETRQQQHIMSEKRI

MGEADCDFVVKLFKTFKDRKYLYMLMEACLGGELWTVLRDKGHFDDGTTRFYTACVVEAF

DYLHSRNIIYRDLKPENLLLDSQGYVKLVDFGFAKRLDHGRKTWTFCGTPEYVAPEVILN

KGHDISADYWSLGVLMFELLTGTPPFTGGDPMKTYNIILKGIDAIEFPRSITRNATALIK

KLCRDNPAERLGYQKGGISEIQKHKWFDGFNWEGLRARTLEPPIMPRVQNATDTTNFDEY

PPDSDPPPPDDISGWDNDF
