# Supplementary material for: Trypanosomes Modify the Behavior of Their Insect Hosts: Effects on Locomotion and on the Expression of a Related Gene
Source: PLoS Negl Trop Dis. 2015 Aug 20;9(8):e0003973. doi: 10.1371/journal.pntd.0003973 (PMC4546274; doi:10.1371/journal.pntd.0003973)
Supplement: S1 Table — Pairwise contrasts were used to evaluate the locomotory activity of uninfected and infected individuals at every hour of the day. P-values of the contrasts were adjusted by Holm-Bonferroni method to correct for the problem of multiple comparisons. (DOCX) [file pntd.0003973.s002.docx]

| **Levels** | **Hour** | **Contrast** | **df** | **Chi square** | **P-value** |
| --- | --- | --- | --- | --- | --- |
| Control *vs.* Infected | 19:00-20:00 | -0.02259 | 1 | 0.0082 | 1.0000 |
| Control *vs.* Infected | 20:00-21:00 | 1.09148 | 1 | 19.0998 | 0.0003 |
| Control *vs.* Infected | 21:00-22:00 | 0.64667 | 1 | 6.7044 | 0.2116 |
| Control *vs.* Infected | 22:00-23:00 | 0.68066 | 1 | 7.4278 | 0.1477 |
| Control *vs.* Infected | 23:00-00:00 | 0.554 | 1 | 4.9206 | 0.5308 |
| Control *vs.* Infected | 00:00-01:00 | 0.62075 | 1 | 6.1777 | 0.2717 |
| Control *vs.* Infected | 01:00-02:00 | 0.40207 | 1 | 2.5917 | 1.0000 |
| Control *vs.* Infected | 02:00-03:00 | 0.48575 | 1 | 3.7828 | 0.9839 |
| Control *vs.* Infected | 03:00-04:00 | 0.20861 | 1 | 0.6977 | 1.0000 |
| Control *vs.* Infected | 04:00-05:00 | 0.2827 | 1 | 1.2813 | 1.0000 |
| Control *vs.* Infected | 05:00-06:00 | 0.08656 | 1 | 0.1201 | 1.0000 |
| Control *vs.* Infected | 06:00-07:00 | 0.13418 | 1 | 0.2887 | 1.0000 |
| Control *vs.* Infected | 07:00-08:00 | -0.1947 | 1 | 0.6077 | 1.0000 |
| Control *vs.* Infected | 08:00-09:00 | -0.10005 | 1 | 0.1605 | 1.0000 |
| Control *vs.* Infected | 09:00-10:00 | 0.08712 | 1 | 0.1217 | 1.0000 |
| Control *vs.* Infected | 10:00-11:00 | 0.11097 | 1 | 0.1974 | 1.0000 |
| Control *vs.* Infected | 11:00-12:00 | 0.08564 | 1 | 0.1176 | 1.0000 |
| Control *vs.* Infected | 12:00-13:00 | 0.05231 | 1 | 0.0439 | 1.0000 |
| Control *vs.* Infected | 13:00-14:00 | 0.04523 | 1 | 0.0328 | 1.0000 |
| Control *vs.* Infected | 14:00-15:00 | -0.15128 | 1 | 0.3669 | 1.0000 |
| Control *vs.* Infected | 15:00-16:00 | 0.07482 | 1 | 0.0898 | 1.0000 |
| Control *vs.* Infected | 16:00-17:00 | 0.04607 | 1 | 0.034 | 1.0000 |
| Control *vs.* Infected | 17:00-18:00 | -0.06223 | 1 | 0.0621 | 1.0000 |
| Control *vs.* Infected | 18:00-19:00 | -0.05365 | 1 | 0.0461 | 1.0000 |
