# Supplementary material for: Trypanosomes Modify the Behavior of Their Insect Hosts: Effects on Locomotion and on the Expression of a Related Gene
Source: PLoS Negl Trop Dis. 2015 Aug 20;9(8):e0003973. doi: 10.1371/journal.pntd.0003973 (PMC4546274; doi:10.1371/journal.pntd.0003973)
Supplement: S2 Table — Pairwise contrasts were used to evaluate the locomotory activity of uninfected and infected individuals at every hour of the day. P-values of the contrasts were adjusted by Holm-Bonferroni method to correct for the problem of multiple comparisons. (DOCX) [file pntd.0003973.s003.docx]

| **Levels** | **Hour** | **Contrast** | **df** | **Chi square** | **P-value** |
| --- | --- | --- | --- | --- | --- |
| Control *vs.* Infected | 19:00-20:00 | 0.22144 | 1 | 0.7185 | 1.0000 |
| Control *vs.* Infected | 20:00-21:00 | 0.25362 | 1 | 0.9425 | 1.0000 |
| Control *vs.* Infected | 21:00-22:00 | -0.23084 | 1 | 0.7808 | 1.0000 |
| Control *vs.* Infected | 22:00-23:00 | -0.32487 | 1 | 1.5464 | 1.0000 |
| Control *vs.* Infected | 23:00-00:00 | -0.4311 | 1 | 2.7232 | 1.0000 |
| Control *vs.* Infected | 00:00-01:00 | -0.43314 | 1 | 2.7489 | 1.0000 |
| Control *vs.* Infected | 01:00-02:00 | -0.40241 | 1 | 2.3728 | 1.0000 |
| Control *vs.* Infected | 02:00-03:00 | -0.44396 | 1 | 2.888 | 1.0000 |
| Control *vs.* Infected | 03:00-04:00 | -0.18502 | 1 | 0.5016 | 1.0000 |
| Control *vs.* Infected | 04:00-05:00 | -0.33765 | 1 | 1.6705 | 1.0000 |
| Control *vs.* Infected | 05:00-06:00 | -0.15547 | 1 | 0.3542 | 1.0000 |
| Control *vs.* Infected | 06:00-07:00 | -0.12282 | 1 | 0.221 | 1.0000 |
| Control *vs.* Infected | 07:00-08:00 | 1.17788 | 1 | 20.3293 | 0.0002 |
| Control *vs.* Infected | 08:00-09:00 | 0.38979 | 1 | 2.2263 | 1.0000 |
| Control *vs.* Infected | 09:00-10:00 | -0.21974 | 1 | 0.7075 | 1.0000 |
| Control *vs.* Infected | 10:00-11:00 | -0.77977 | 1 | 8.9095 | 0.0596 |
| Control *vs.* Infected | 11:00-12:00 | -0.62862 | 1 | 5.7903 | 0.2901 |
| Control *vs.* Infected | 12:00-13:00 | -0.55111 | 1 | 4.4504 | 0.5583 |
| Control *vs.* Infected | 13:00-14:00 | -0.46803 | 1 | 3.1743 | 1.0000 |
| Control *vs.* Infected | 14:00-15:00 | -0.57323 | 1 | 4.7617 | 0.4947 |
| Control *vs.* Infected | 15:00-16:00 | -0.73385 | 1 | 7.3452 | 0.1278 |
| Control *vs.* Infected | 16:00-17:00 | -0.80219 | 1 | 8.777 | 0.0610 |
| Control *vs.* Infected | 17:00-18:00 | -0.98651 | 1 | 13.2739 | 0.0059 |
| Control *vs.* Infected | 18:00-19:00 | -1.0219 | 1 | 14.2433 | 0.0037 |
